# Genetic feature selection algorithm as an efficient glioma grade classifier

**DOI:** 10.1038/s41598-024-83879-2

**Published:** 2025-05-03

**Authors:** Ting-Han Lin, Hung-Yi Lin

**Affiliations:** 1 China Medical University Hospital, No. 2, Yude Rd., North Dist., Taichung City, 404327 Taiwan; 2 Department of Distribution Management, National Taichung University of Science and Technology, 129, Sanmin Rd., Sec. 3, Taichung, Taiwan (R.O.C.)

**Keywords:** Glioma, Genetic testing, Gene selection, Computational analysis, Molecular analysis, Heuristic feature selection, Classification efficiency, Discretization, Biomarkers, Molecular medicine

## Abstract

Gliomas are among the most lethal and debilitating cancers. Genetic testing is a rapidly evolving modality for cancer management. The advent of DNA microarrays enabled the utility of computational analyses in such management on a molecular basis. However, as current computational analyses remain insensitive to interactions between molecular features, they rarely postulate reasonable pathogenesis. The current study proposes a heuristic feature selection algorithm that identifies subsets of genes to almost perfectly classify glioma grades. The discretization technique in our method is a powerful tool against the tremendous data volume in DNA microarray. Instead of recognizing individual genetic features, the proposed algorithm helps identify specific gene subsets that play important roles in the pathogenesis of glioma.

## Introduction

Despite decades of investigation, brain tumors remain among the most lethal and debilitating malignancies of all forms^1^. Glioma, the most common variant of brain tumors in the adult population, has emerged as the subject of intensive research over the past three decades^2^. Tumor grading, or the ability to determine the biological aggressiveness of a tumor, is a pivotal element in treatment planning, disease monitoring and prognosis for those afflicted. However, manual identification by oncologists and radiologists is a time-consuming and error-prone process. On the other hand, molecular insight into tumor heterogeneity has become increasingly accepted as the key to personalized medicine and advanced cancer treatments.

With the advent of large-scale transcriptional profiling such as DNA microarrays and convenient access to their databases, numerous studies are now able to identify molecularly distinct cancer subtypes, including those of breast cancer^3^ and lung adenocarcinoma^4^, among patients with identical histopathological typing. Advances can also be observed in cancer prediction^5^ and prognosis^6^ with the aid of computational gene analyses. As tumor grading is moving from a morphology-based maneuver to a molecular-based system^2^, feature selection with classification efficiency emerges as an outstanding strategy for biomedical researchers.

For glioma, the development and understanding of its genomic landscape have yielded internally consistent molecular classifiers^7^ as well as computational development of prognostic stratification algorithms^8^. For instance, Zhao et al. uncovered several key genes that might play critical roles in the progression from low-grade glioma to the more malignant form of secondary glioblastoma (GBM)^9^. On the other hand, with the aid of principal component analysis, Cai et al. indicated that Gβγ heterodimers may correlate with the subgrouping of glioma patients^10^. In this regard, these genes may serve as diagnostic and prognostic biomarkers, as well as potential therapeutic targets for glioma. Nevertheless, there exists an incomprehensiveness in the searching process for discriminative gene, and as current computational analyses often neglect interactions between molecular features, they can rarely postulate reasonable pathogenesis based on the calculations. We observe that an exhaustive application of such technique in glioma grading is still lacking.

As a result, to provide a computer-based analysis for future practice, the current study proposes a discretization-based genetic feature selection algorithm as an efficient glioma grade classifier.

## Methods

### Gene expression preprocessing and gene evaluation

The measure of gene expression levels depends on many factors. Uncertainties arise from different situations such as device calibration, probing environment (temperature, humidity, brightness), hybridization of samples or chips, and so forth. Direct use of raw expression profile in analytical procedure is plagued by much impurity and controversial information, which tend to confuse the learning model. Adequate data preprocessing intensifies the valuable information so as not to blur the pivotal elements^11^.

Cluster analysis (CA) is responsible for grouping a set of objects in such a way that objects in the same cluster are more like one another than to those in the other clusters. CA is used in the current study for the discretization task of gene expression preprocessing. Gene expression analysis should focus more on samples with dissimilarities than those with similarities. The measured gene expressions in real-values are suggested to be clustered into groups. Then, only the differences in the distinct clusters are taken into consideration in the subsequent analytic processing.

Hard clustering methods, such as hierarchical clustering and K-means clustering, are widely applied in the early stages of microarray analysis. Fuzzy clustering algorithm allows each data point to belong to more than one cluster. Since conditionally coregulated or co-expressed genes are identified^12^, fuzzy clustering is more appropriate than hard clustering as sample-based clustering is taken as a necessary process. EM is distribution-based clustering algorithm capable of capturing correlation and dependence between data. EM speculates the data distribution of gene expression data, as the expectation step is processed with the implicit underlying variables with an aim to generating estimates of explicit parameters. EM can iterate and then converge the statistical model with latent variables to find the maximum likelihood of the observed samples. EM is particularly suitable in cases where models or systems involve latent variables, unknown parameters, and known data observations. EM is applied to the expression levels of a single gene, and the discretized results are used to evaluate the discrimination power for every single gene.

Shannon’s *information theory* quantifies the uncertainty involved in the prediction of the value of a random variable. The most important type of uncertainty is entropy, which measures the amount of uncertainty associated with a discrete random variable. Information Gain (*IG*)^13^ is an entropy-based way of comparing two distributions. The KL-divergence from a distribution$\:\:p\left(x\right)$ to a distribution $\:q\left(x\right)$ can be thought of as a distance measured from the variable *P* to *Q*,

1 $$\:KL\left(p|q\right)=-\sum\:{p\left(x\right)log}_{2}\left(\frac{q\left(x\right)}{p\left(x\right)}\right).$$

From the perspective of information theory, information gain is defined as the KL-divergence from the observed joint distribution of *X* and *Y* to the product of their observed margin.

2 $$\:\:IG\left( {x,y} \right) = KL\left( {p\left( {x,y} \right)|p\left( x \right)p\left( y \right)} \right) = - \sum {\:_{{x \in X}} } \sum \: _{{y \in Y}}^{{}} p(x,y)\log _{2} \left( {\frac{{p\left( x \right)p\left( y \right)}}{{p(x,y)}}} \right).$$

Information Gain is the typical filter criterion commonly used in feature ranking and relevance analysis. In the current study, the clustering analysis of EM along with *IG* helps to evaluate the sole discrimination effect for every single gene. Then the clustering analysis of FCM along with *IG* helps to evaluate the combined discrimination effect for multiple selected genes.

### Method for approving multiple informative genes

This section proposes the mechanism for approving the effectiveness of add-on genes. That is the core of our algorithm, which is responsible for authenticating the new informative genes for current subsets of genes.

Suppose genes *A* and *B* respectively have *n* and *m* distinct expression values, i.e., *a*_*i*_ and *b*_*j*_, where 1 ≤ *i* ≤ *n* and 1 ≤ *j* ≤ *m*. The dataset with a target class label *C* classified by gene *A* will result in *n* subsets. In other words,

$\:C={C}_{A={a}_{1}}\cup\:{C}_{A={a}_{2}}{\cup\:\dots\:\cup\:C}_{A={a}_{n}}$. Similarly, *C* classified by gene *B* results in *m* subsets, i.e., $\:C={C}_{B={b}_{1}}\cup\:{C}_{B={b}_{2}}{\cup\:\dots\:\cup\:C}_{B={b}_{m}}$. The information entropy from *C* is denoted as *H*(*C*). The information entropy from *C* classified by *A* is denoted as $\:H\left(C\right|A)$ and formulated as following

3 $$\:H\left(A\right)={\sum\:}_{i=1}^{n}\frac{\left|{C}_{A={a}_{i}}\right|}{\left|C\right|}\times\:H\left({C}_{A={a}_{i}}\right).$$

Information gains$\:\:IG\left(C,A\right)$ and $\:IG\left(C,A\cup\:B\right)$ are respectively calculated by $\:H\left(C\right)-H\left(C\right|A)$ and$\:\:H\left(C\right)-H\left(C\right|A\cup\:B)$.

On the other hand, it assumes gene *A* is already in use and then gene *B* is going to join the classification task. The corresponding information entropy when *A* and *B* are taken will become

4 $$\:H\left(A\cup\:B\right)={\sum\:}_{i=1}^{n}{\sum\:}_{j=1}^{m}\frac{\left|{C}_{A={a}_{i},B={b}_{j}}\right|}{\left|C\right|}\times\:H\left({C}_{A={a}_{i},B={b}_{j}}\right).$$

Hence, the improvement of effect derived from gene *B* can be evaluated by following deduction:

$$\:IG\left(C,A\cup\:B\right)-IG\left(C,A\right)=H\left(C\right)-H\left(A\cup\:B\right)-[H\left(C\right)-H(C\left|A\right)]$$

5 $$\:=H\left(C\right|A)-H\left(A\cup\:B\right)$$

This improvement is denoted as $\:\varDelta\:IG\left(B|A\right)$. Extensively, in the case of a collection of genes (denoted as *S*) has already been taken, the effect improvement caused by a newly added gene $\:\alpha\:$ can be evaluated by $\:\varDelta\:IG\left(\alpha\:|S\right)=IG\left(C,FCM(S\cup\:\alpha\:)\right)-IG\left(C,FCM\left(S\right)\right)$, where $\:S\cup\:\alpha\:$ and$\:\:S$ are respectively discretized into categorical data by fuzzy c-means (FCM). As a result, the higher $\:\varDelta\:IG\left(\alpha\:|S\right)$ boosts the discrimination power of $\:S\cup\:\alpha\:$ to a higher level and validates the necessity of $\:\alpha\:$. FCM developed by Dunn^14^ and improved by Bezdek^15^ is a well-known soft clustering algorithm that allows one piece of data to belong to two or more clusters. This method is different from the hard clustering algorithm in which each object strictly belongs to exactly one cluster. FCM simultaneously handles the expression levels of several genes, and the results facilitate the evaluation of such a set of genes. By applying FCM, we aim to largely identify the similar and dissimilar gene vectors for the discretization of combined genes. In this way, we would be able to discretize several genes acted on by more than one transcription factor and encode multi-functional proteins.

### Genetic feature selection algorithm

We propose a three-stage algorithm. In the first stage, all genes are analyzed by EM and evaluated by *IG*. This stage aims to quickly filter the irrelevant genes. Then, instead of taking all genes as candidates, the second stage only collects a limited number of relevant genes in the set *U* and in turn the best *N* genes in *U* are taken as the starts of different heuristic selections. The third stage is the iterative process for heuristic learning based on the *N* genes. The notations used in our heuristic algorithm are explained as follows:

- *U*, *K*: The set contains all candidate genes and the cardinality of *U* is *K*.
- $\:{A}_{1}$, *N*: The set contains the top genes in *U* and the cardinality of $\:{A}_{1}$ is *N*.
- $\:{A}_{i}$: The set contains the combinatorial subsets which are hybrid from *i* genes, *i* = 2,3,….
- $\:S$: The set contains the authenticated genes.
- $\:C$: Classification outcome (e.g., diagnostic outcome).

**Fig. 1:**
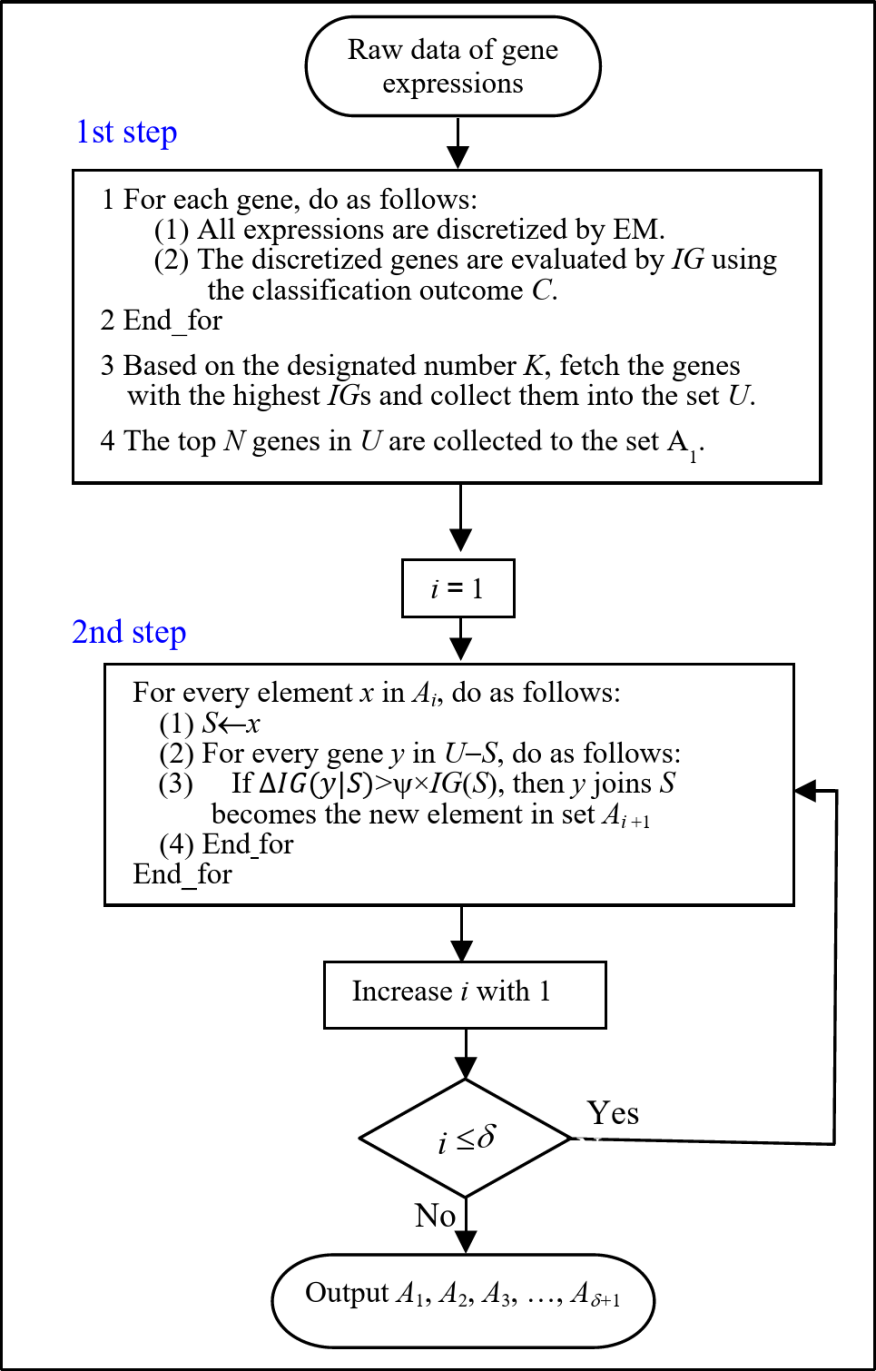
Flow chart of designs.

We propose a two-step genetic feature selection algorithm as shown in Fig. 1. In the first step, every raw gene is analyzed and preprocessed by the EM for discrimination. Then, a few *K* candidate genes are preserved. The designated value of *K* is based on the search overheads spent. Percentages of 1%~10% of the number of raw genes could be reasonable choices. In this study, 1.5% (around 300 genes) are assigned to *K*. This step aims to primarily and quickly distinguish the relevant and irrelevant genes based on their *IG* evaluations. And then, a number of *N* top genes are collected as the seeds for subsequent heuristic searching. The value of *N* depends on the desired width of selection. The bigger the *N* is, the more widely the search paths would be launched. In this study, we assign that *N* equals 5.

For the second step, we propose a loop procedure controlled by two parameters of *ψ* and *δ*. Parameter *ψ* is responsible for regulating the improved threshold of *IG* evaluation and determining the width of selection, while *δ* is responsible for the depth of selection. Initially, *i* equals 1 as the first searching round is activated. Every single gene in $\:{A}_{1}$ serves as the starter with which we will attempt to match other candidates. Since *N* is assigned as 5, five searching directions and hundreds of matchings are triggered. $\:{A}_{2}$ collects the paired genes in cases that the tested gene *y* can improve *IG*(*S*) over the given percentage (i.e., ψ*100%). The value of *ψ* could be floating. For example, in earlier periods, we apply a loose setting of *ψ* in an attempt to collect plentiful results, while we adjust its value to a stricter setting for narrowing the subsequent results in the following periods. The parameter *δ* determines the depth of selection. In this study, we set a maximum of *δ* to be 9 to generate $\:{A}_{10}$. That is, the farthest searches, in the setting of δ being 9, results in a subset with 10 gene members unless *IG*(*S*) ceases to be improved or the improvement is below the threshold of *ψ*. Finally, $\:{A}_{1}$ outputs five single genes, while $\:{A}_{2}$ and $\:{A}_{3}$ respectively output a lot of paired and trio genes. In general, $\:{A}_{i}$ outputs a bundle of gene(s) with a size of *i* gene(s).

For different datasets, our algorithm could assign different values of *K*, *N*, *ψ*, and *δ*. Even in one single dataset, respective requirements of different experiments might lead to variable assignments. In order to guide the selection process, we suggest the following parameter setting. The design of our heuristic algorithm is tailor-made for large-scale datasets with high feature space, especially those with microarray data. Hence, the value of *K* for the candidate gene number is limited to 1%~10% of the total number of raw features. Furthermore, to prevent from activating the exponential number of searching paths in our heuristic method, the parameters *N*, *ψ*, and *δ* are set as following. The value of *N* is limited to a low but enough amount between $\lfloor\:\text{ln}K\rfloor$ to $\lfloor\:2\cdot\text{ln}K\rfloor.$ The greater the *N* is, the higher number of candidate features there will be. Parameter *ψ* is responsible for regulating the improved threshold of *IG* and decides the searching width. It must be nonnegative, and we suggest that *ψ* = 1%~3%. A greater *ψ* value can restrict the generated number of searching paths. The maximal selection round (i.e., depth of the searching process) is confined by *δ*. It means our algorithm can accordingly select the maximal size of (*δ* + 1) genes into the set *A*_*δ +* 1_. Theoretically, we suggest the estimation of $\lfloor\:\text{ln}M\rfloor$ to be the assignment of *δ*, where *M* is the total feature number. As a result, we assign values of *K* = 300, *N* = 5, *ψ* = 0.02, and *δ* = 9 in this study to differentiate grade III and IV glioma. The experimental results will be discussed in the next section.

## Results

### Dataset acquisition

The dataset we utilized in this study is Gene Expression Omnibus (GSE) 4412, titled “freij-affy-human-91666”^16^. Transcriptional profiling has been applied to 85 glioma samples from 74 patients to investigate glioma biology, prognosticate survival, and define tumor subclasses. Patients participating in this broad protocol were analyzed if their initial tumor was diagnosed as a grade III or IV glioma of any histologic type on initial surgical treatment. Only grade III and IV gliomas were included in this study as the distinction between these two is subtle and prone to misclassification. The time in days elapsed from resection to the day of death, or if the patient has remained alive, to the study endpoint was recorded for all samples studied.

The characteristics of the dataset are listed in Table 1. 85 samples of 74 patients were collected in this broad protocol. Only grade III and IV gliomas were included in this study. A number of 26 grade III samples from 20 patients and a number of 59 grade IV samples from 50 patients were obtained. Patient ages at diagnosis spanned from 18 to 82 years. There were 46 females and 28 males. For the implementation of our proposed algorithm, the gene identifiers are simply re-coded by the sequential number from V1 to V22283. All data preprocessing, cluster analysis, gene evaluation, and gene selection were implemented in R programming languages executed on a workstation with an Intel core i5 CPU 3.00 GHz and 8.00 GB RAM.

**Table 1:** Dataset characteristics.

Dataset	Number of genes	Number of samples/patients	Number of classes (grade III/IV)
GSE4412	22,283	85/74	2 (26/59)

### Gene selection

The 10 selection rounds produced 10 output sets $\:{A}_{1}$~$\:{A}_{10}$, whose best-performing components have been listed in Table 2. The generated sets and their best-performing component(s) are shown in the first two columns. The evaluated *IG* for each best component is shown in the third column. The information entropy from the classification outcome *C* of the 85 samples is $\:H\left(C\right)={-\left(\frac{26}{85}\right)\times\:\text{log}}_{2}(\frac{26}{85}){-\left(\frac{59}{85}\right)\times\:\text{log}}_{2}\left(\frac{59}{85}\right)\:\cong0.8884$. This value indicates the maximal inherent information amount and could be the upper bound for discrimination capability of gene(s). In this study, the classification effectiveness (abbreviated as *CE*) of one selected gene subset *S* is measured as $\:IG\left(S\right)/H\left(C\right)\times\:100{\%}$. A perfect *CE* of 100% suggests that *S* is able to classify the exact grades (i.e., III or IV) without any error. As shown in the last column of Table 2, our proposed method heuristically selects genes with continuous *CE* growth. We observed that significant improvement occurred during the first five rounds and then stayed at the high level of around 92% until the 10th round. The corresponding gene identifiers and encoded proteins of these selected genes in NCBI Dataset GSE4412 are listed in Table 3. In our Discussion section, we will discuss further on how these proteins relate to the pathogenesis of glioma.

Based on our experiment design, the gene labelled as V3455 was identified as the best-performing component in $\:{A}_{1}$. However, using V3455 with *IG* value of 0.4706 we can only achieve the *CE* of 52.97%. This reveals that about 52.97% of information among 85 samples can be discriminated out by V3455. As we proceeded to the second round, after examining every gene in the set *U*, no gene can assist V3455 in achieving a better *IG* value, and thus we halt this searching path. Using other genes in $\:{A}_{1}$ to trigger different searching paths, paired-genes with better *IG* values are included into $\:{A}_{2}$. Our results indicated that V1328 in $\:{A}_{1}$ advances the searching path, and we collected all the genes that benefit V1328 into $\:{A}_{2}$. Among all pairs in $\:{A}_{2}$, (V1328, V4573) has the best performance according its *IG* value. Precisely, (V1328, V4573) gained an improved *IG* of 0.5989 and its corresponding *CE* of 67.42% (shown in the second row of Table 2). Note that V3455 is no longer involved, indicating that our algorithm does not necessarily inherit its previous output, but rather values the enhancement of the overall effect.

Proceeding to the third round, the similar selection logic is applied. Based on V1328 and V4344, V16253 was recognized as best next gene among all candidate genes in set *U*. Successfully, the *IG* has advanced to 0.6878 and the achieved *CE* is 77.42%. For the 4th and 5th rounds, the best components in A_4_ and $\:{A}_{5}$ were seen to continuously improve their performance. For $\:{A}_{5}$, the *IG* and *CE* are 0.8228 and 92.62%, respectively. We note that the searching paths anchored on V1328 and V12974 all the way to the 10th round. On the molecular basis, V1328 is responsible for producing interleukin 1 (IL-1) receptor type 2 and is possibly linked to the formation of tumor microenvironment. On the other hand, V12974 represents the gene of Zinc Finger CCCH-Type Antiviral Protein 1 (ZC3HAV1). The detailed effect and pathogenesis about the selected genes will be explained in the Discussion section. After the 5th round, marginal improvement can still be observed. In this study, $\:{A}_{10}$ reaches a high of 92.73% in *CE*, which almost perfectly classifies glioma grades.

In the 6th to 10th rounds, the best components are based on the ones found in the 5th round. As a result, it would be enlightening to understand the functionality of the five genes in $\:{A}_{5}$, which we will look deeper into in the Discussion section (see Table 3, where the corresponding gene identifiers of the best-performing component(s) in $\:{A}_{1}$-$\:{A}_{10}$ and their encoded protein(s) are shown).

**Table 2:** The best-performing component(s) in $\:{A}_{1}$-$\:{A}_{10}$.

Round (i)	Best-performing component in $\:{\varvec{A}}_{\varvec{i}}$	Discrimination power (IG)	Classification effectiveness (CE)
1	V3455	0.4706	52.97%
2	V1328∪V4573	0.5989	67.42%
3	V1328∪V4344∪V16253	0.6878	77.42%
4	V1328∪V12974∪V19735∪V22018	0.7730	87.01%
5	V1328∪V12974∪V14901∪V20441∪V22030	0.8228	92.62%
6	V1328∪V12974∪V14901∪V20441∪V22030∪V22042	0.8228	92.62%
7	V1328∪V12974∪V14901∪V20441∪V22030∪V22042∪V22058	0.8228	92.62%
8	V1328∪V12974∪V14901∪V20441∪V22030∪V22042∪V22058∪V22065	0.8228	92.62%
9	V1328∪V12974∪V14901∪V20441∪V22030∪V22042∪V22058∪V22104∪V22193	0.8238	92.73%
10	V1328∪V12974∪V14901∪V20441∪V22030∪V22042∪V22058∪V22104∪V22193∪V22195	0.8238	92.73%

**Table 3:** The corresponding gene identifiers of the best-performing component(s) in $\:{A}_{1}$-$\:{A}_{10}$ and their encoded protein(s).

Round	Gene identifier	Encoded protein
1	(203388_at)	[arrestin beta 2]
2	(211372_s_at)∪(204506_at)	[interleukin 1 receptor type 2] ∪[protein phosphatase 3 regulatory subunit B, alpha]
3	(211372_s_at)∪(204277_s_at)∪(216342_x_at)	[interleukin 1 receptor type 2] ∪[thymidine kinase 2, mitochondrial] ∪[Ribosomal Protein S4 X-Linked]
4	(211372_s_at)∪(213051_at)∪(219831_at)∪(222118_at)	[interleukin 1 receptor type 2] ∪[zinc finger CCCH-type containing, antiviral 1] ∪[cyclin dependent kinase like 3] ∪[centromere protein N]
5	(211372_s_at)∪(213051_at)∪(214986_x_at)∪(220537_at)∪(222130_s_at)	[interleukin 1 receptor type 2] ∪[zinc finger CCCH-type containing, antiviral 1] ∪[peptidylprolyl isomerase like 2] ∪[ankyrin repeat and SOCS box containing 12] ∪[mitochondrial rRNA methyltransferase 2]
6	(211372_s_at)∪(213051_at)∪(214986_x_at)∪(220537_at)∪(222130_s_at)∪(222142_at)	[interleukin 1 receptor type 2] ∪[zinc finger CCCH-type containing, antiviral 1] ∪[peptidylprolyl isomerase like 2] ∪[ankyrin repeat and SOCS box containing 12] ∪[mitochondrial rRNA methyltransferase 2] ∪ [CYLD lysine 63 deubiquitinase]
7	(211372_s_at)∪(213051_at)∪(214986_x_at)∪(220537_at)∪(222130_s_at)∪(222142_at)∪(222158_s_at)	[interleukin 1 receptor type 2] ∪[zinc finger CCCH-type containing, antiviral 1] ∪[peptidylprolyl isomerase like 2] ∪[ankyrin repeat and SOCS box containing 12] ∪[mitochondrial rRNA methyltransferase 2] ∪ [CYLD lysine 63 deubiquitinase] ∪ [desumoylating isopeptidase 2]
8	(211372_s_at)∪(213051_at)∪(214986_x_at)∪(220537_at)∪(222130_s_at)∪(222142_at)∪(222158_s_at)∪(222165_x_at)	[interleukin 1 receptor type 2] ∪[zinc finger CCCH-type containing, antiviral 1] ∪[peptidylprolyl isomerase like 2] ∪[ankyrin repeat and SOCS box containing 12] ∪[mitochondrial rRNA methyltransferase 2] ∪ [CYLD lysine 63 deubiquitinase] ∪ [desumoylating isopeptidase 2] ∪ [chromosome 9 open reading frame 16]
9	(211372_s_at)∪(213051_at)∪(214986_x_at)∪(220537_at)∪(222130_s_at)∪(222142_at)∪(222158_s_at)∪(222204_s_at)∪(222294_s_at)	[interleukin 1 receptor type 2] ∪[zinc finger CCCH-type containing, antiviral 1] ∪[peptidylprolyl isomerase like 2] ∪[ankyrin repeat and SOCS box containing 12] ∪[mitochondrial rRNA methyltransferase 2] ∪ [CYLD lysine 63 deubiquitinase] ∪ [desumoylating isopeptidase 2] ∪ [null]* ∪[Ras-related protein Rab-27a]
10	(211372_s_at)∪(213051_at)∪(214986_x_at)∪(220537_at)∪(222130_s_at)∪(222142_at)∪(222158_s_at)∪(222204_s_at)∪(222294_s_at)∪(222296_at)	[interleukin 1 receptor type 2] ∪[zinc finger CCCH-type containing, antiviral 1] ∪[peptidylprolyl isomerase like 2] ∪[ankyrin repeat and SOCS box containing 12] ∪[mitochondrial rRNA methyltransferase 2] ∪ [CYLD lysine 63 deubiquitinase] ∪ [desumoylating isopeptidase 2] ∪ [null]* ∪[Ras-related protein Rab-27a] ∪[null]*

*Encoded proteins of (222204_s_at) and (222296_at) lack description in the dataset GSE4412.

To address the effectiveness of the individual selected genes, we conduct SHAP value evaluations over the best-performing components for the 3rd, 5th, 7th, and 9th rounds. First, the greater evaluation of 「Mean |SHAP value| 」in V1328 tells that it is the most important component gene in our experimental results and whose low expression values (i.e., low feature values) can cause high 「SHAP value」 and in turn possesses the better discriminative effect on grade III and IV glioma. This finding is consistent with our experimental results of *IG* and *CE*. And such condition continues to be remarkable until the 9th round. In addition, V12974 is the critical gene which can particularly enhance the discrimination power of V1328 starting from the 4th round. As shown in Table 4, V12974 possesses almost the half influence of V1328 on the classification task. Unlike V1328, the explanation of expression values of V12974 is proportional to the impact degree. That is, the higher feature values have the greater impact on the classification model. Since V1328 and V12974 are selected in the 4th round, their combined effects dominate the discrimination task in the selection rounds thereafter and ensure the high classification performance.

**Table 4:** The individual performance of the selected genes in different rounds.

Round	Mean |SHAP value| and SHAP value
3	

5	

7	

9	

## Discussion

Modern data management systems have no lack of information but a dearth of practical applicability. Mining useful knowledge is the philosophy of integrating diverse disciplines and technologies. The current study discriminates between glioma grade III and IV with a discretization-based selection algorithm, and its main contributions are twofold. First, discretization using fuzzy c-means enhances the discriminative power of genetic feature selection. Second, instead of recognizing individual genetic features, our heuristic algorithm helps identify specific gene subsets that play fundamental roles in the pathogenesis of glioma.

If we take only one gene into account in $\:{A}_{1}$, the gene with identifier 203388_at (designated as V3455 herein) has the best performance on glioma grading of 52.97%, with an *IG* measurement of 0.4706. V3455 relates to the gene of Arrestin Beta 2 (Arrb2), which is highly expressed in the central nervous system. Low Arrb2 expressions were shown to correlate with high HIF-1α expressions and poor GBM patient survival^17^. However, such grading prediction with a single factor is quite primitive, and the accuracy level remains questionable.

For sets $\:{A}_{2}$ to $\:{A}_{4}$, the effectiveness was seen to continuously improve. Remarkably, in these three sets, V1328 did stand out as the one single gene that was constantly selected. V1328 is responsible for producing interleukin 1 (IL-1) receptor type 2. IL-1 is involved in the modulation of the glioma microenvironment, with implications for increased GBM progression^12,18^. Our algorithm’s identification of V1328 as the pivotal factor to evaluate for other gene additions can be verified by the emerging evidence on the tumor microenvironment bearing key importance in tumorigenesis^19^.

Apart from V1328, the other members of $\:{A}_{2}$ to $\:{A}_{4}$ were too variable for us to make interpretations on a genetic basis. This resonates with our emphasis on the interaction between expressions of data, $\:\varDelta\:IG\left(\alpha\:|S\right)$, instead of focusing on every single selected gene. By contrast, starting from the fifth selection round with set $\:{A}_{5}$, although only marginal improvements were observed in higher rounds, their components were found to be built based on those five, including V1328, already in $\:{A}_{5}$. As a result, it would be reasonable to look deeper into the five genes in $\:{A}_{5}$ (Table 3).

The functionality of V1328 has been discussed in the previous section. 213051_at (i.e., V12974) represents the gene of Zinc Finger CCCH-Type Antiviral Protein 1 (ZC3HAV1). Through bioinformatics analysis and immunoprecipitation assays, Huang et al. reported that ZC3HAV1 is in relation to poor prognosis and metastasis of pancreatic cancer^20^. 214986_x_at (i.e., V14901) is the gene responsible for encoding peptidylprolyl isomerase like 2 (PPIL2), as tissue sample analysis from breast cancer patients showed a significant correlation between PPIL2 expression and the degree of cancer invasion and metastasis^21^. PPIL2 has also been recognized as an effector of the JAK2-STAT5 pathway^22^, while hyperactive JAK/STAT signaling was suggested as the common characteristic of myeloproliferative neoplasms^23^. As for 220537_at (V20441), it is indicative of the gene ASB 12 (ankyrin repeat and SOCS box containing 12). As ASB12 is thought to be possibly linked to endometrial cancer^24^, it was also proven as an oncogene by Chen et al.^25^, upregulating cell proliferation and inhibiting cell cycle arrest. However, the clear connection between ASB12 and glioma is yet to be established. Last but not least, V22030 represents the gene mitochondrial rRNA methyltransferase 2 (MRM2). In a glioma risk prediction formula proposed by Wang et al. in 2021, MRM2 and TRMT2B (tRNA methyltransferase 2 homolog B) are indicated as the primary predictors of the prognosis and clinicopathologic features for glioma patients^26^. In this regard, we suggest that all components in $\:{A}_{5}$ are in connection to malignant formation, with two of them raising direct concern for glioma. Further studies are expected to help explain the functionality of the other genes that are currently not directly linked to glioma.

In addition, the *CE* was observed to plateau from $\:{A}_{5}$ to $\:{A}_{8}$ (92.62%) and then with a slight increase from $\:{A}_{8}$ to $\:{A}_{9}$ (92.62–92.73%) before plateauing again at A_10_. The inclusion of gene V22193 in $\:{A}_{9}$ may be related to the improvement from $\:{A}_{8}$ to $\:{A}_{9}$. V22193 represents the gene RAB27A, member RAS oncogene family, which encodes the Ras-related protein Rab-27a. In a mouse glioma study^27^, Rab27a was reported to take part in glioma cells’ expression of CCL2 and viability in vitro. The same study also suggested that Rab27a has a vital role in glioma cells’ release of small extracellular vesicles. As a result, the fact that the inclusion of gene V22193 improved the *CE* is theoretically compatible with the pathogenesis of glioma.

In the clinical setting, apparatuses equipped with our algorithm can be incorporated into the workflow of pathology laboratories. In the development of gene-specific treatment strategies for populations of different gender, age, ethnicity, etc., our algorithm helps identify the target genes efficiently from various tissue samples. In this regard, our algorithm is expected to provide significant assistance in clinical studies performed by institutions such as medical centers.

The current study has some limitations. First, although our algorithm is capable of pointing out several genes predictive of higher-grade gliomas, further studies will be required to illuminate whether it is the up- or down-regulation of the genes that would help classify glioma grades. Second, for the genes V12974, V14901, V20441 in $\:{A}_{5}$, although they have been selected in our heuristic algorithm, they would currently serve only as observational evidence since their direct linkage to glioma tumorigenesis still requires elucidation.

In conclusion, the current study proposed a discretization-based genetic feature selection algorithm to make efficient classification on glioma grading. It highlights the emerging role of computational analysis in genetic testing for oncology, advances our molecular understanding of glioma, and casts light on the possible directions for future biomedical research.

## Data Availability

The dataset supporting this study’s findings is openly available in NCBI Gene expression Omnibus at https://www.ncbi.nlm.nih.gov/geo/query/acc.cgi? acc=GSE4412.
